# Supplementary material for: A Systemic and Integrated Analysis of p63-Driven Regulatory Networks in Mouse Oral Squamous Cell Carcinoma
Source: Cancers (Basel). 2023 Jan 10;15(2):446. doi: 10.3390/cancers15020446 (PMC9856320; doi:10.3390/cancers15020446)
Supplement: Supplementary file 1 [file cancers-15-00446-s001.zip › MDPI Supplementary Figures Finalized.pdf]

Supplementary Figure 1: Principal component analysis (PCA) of mouse 4NQO-induced oral squamous cell carcinoma cell lines. Top 2500 variable genes were used to generate PCA.

Supplementary Figure 2: qRT-PCR analysis of a selection of DEGs identified in B7E11 p63 knockdown cell line.

Supplementary Figure 3: qRT-PCR analysis of a selection of DEGs identified in B7E3 p63-inducible cell line.

Supplementary Figure 4: Boxplot of expression levels of the genes that constitute the combined p63-based mOSCC gene expression signature in the human TCGA HNSCC cohort.

Supplementary Figure 5: Kaplan-Meier survival analysis of the genes that constitute the combined p63-based mOSCC gene expression signature in the human TCGA HNSCC cohort.

Supplementary Figure 6: Immunofluorescent staining of COTL1 shows novel nuclear staining in areas of low DNA density, except for actively dividing cells in which difused COTL1 expression is observed surrounding the condensed DNA. Yellow arrows highlight the punctual staining of COTL1, while white arrows highlight the diffuse staining of COTL1.

Supplementary Figure 7: Full Western blots of parental mouse 4NQO-induced cell lines B7E3, B4B8, and B7E11 as seen in Figure 1.

Supplementary Figure 8: Full Western blots of B7E11 p63 knockdown cell line as seen in Figure 3.

Supplementary Figure 9: Full Western blots of B7E3 inducible p63 cell lines as seen in Figures 4 and 6.

Supplementary Table 1: Combined Table showing results of mOSCC cell line DEG analyses.

Supplementary Table 2: B7E11 p63 ChIP-seq Combined Sites.

Supplementary Table 3: B7E11 p63 knockdown DEG list.

Supplementary Table 4: B7E11 p63 inducible DEG list.

Supplementary Table 5: Combined loss- and gain-of-function p63-based mOSCC Signature.

Supplementary Table 6: 4NQO-induced mOSCC DEG analysis.

Supplementary Table 7: Combined overall p63-based gene expression signature.

Supplementary Table 8: scRNA-seq DEG analysis based on p63high versus p63low cell populations.

Supplementary Table 9: List of primers utilized in qRT-PCR analysis.

Figure S1

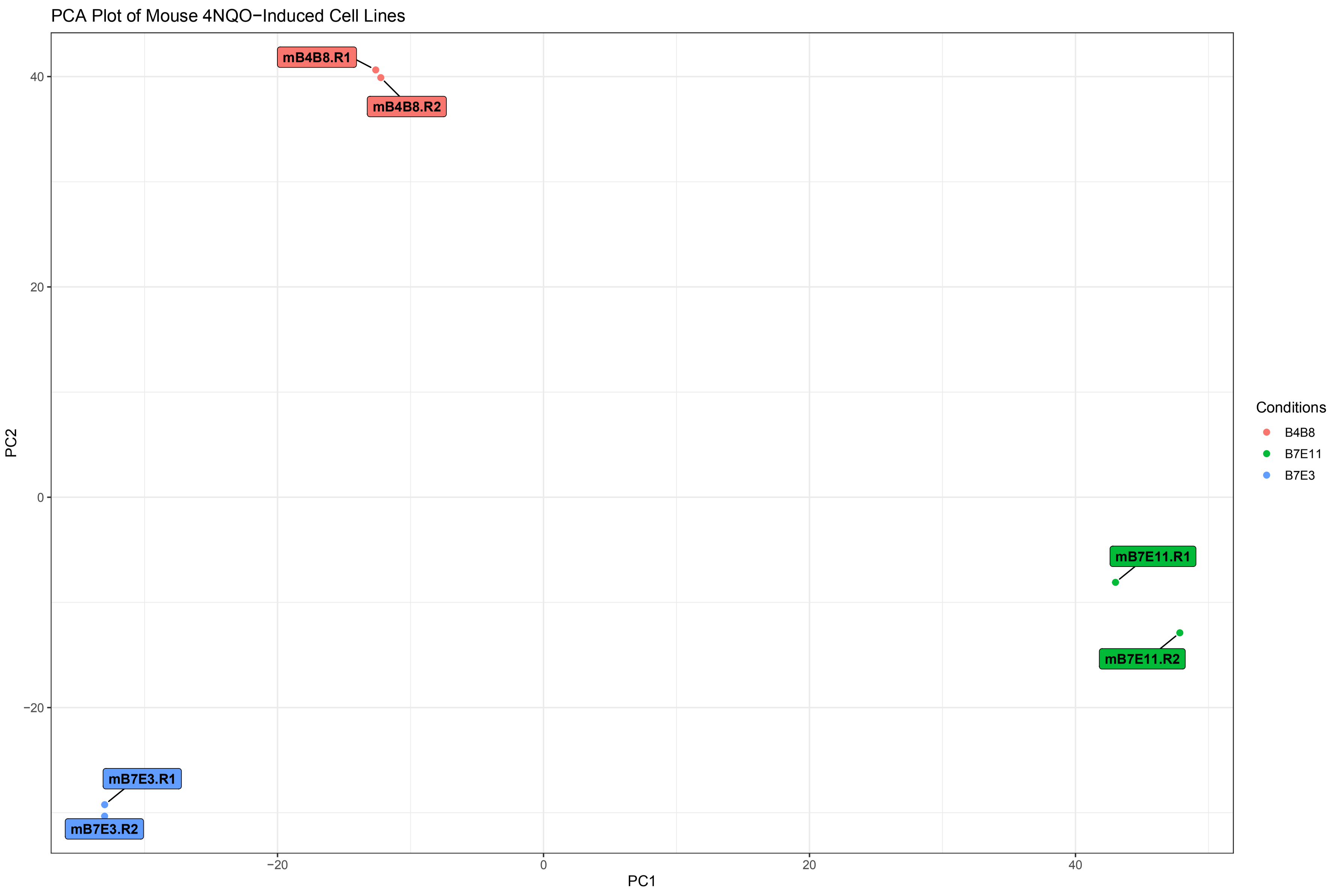

PCA Plot of Mouse 4NQO−Induced Cell Lines
mB4B8.R1
mB4B8.R2
mB7E11.R1
mB7E11.R2
mB7E3.R1
mB7E3.R2
40
20
0
−20
PC2
−20
0
20
40
PC1
Conditions
B4B8
B7E11
B7E3

Figure S2

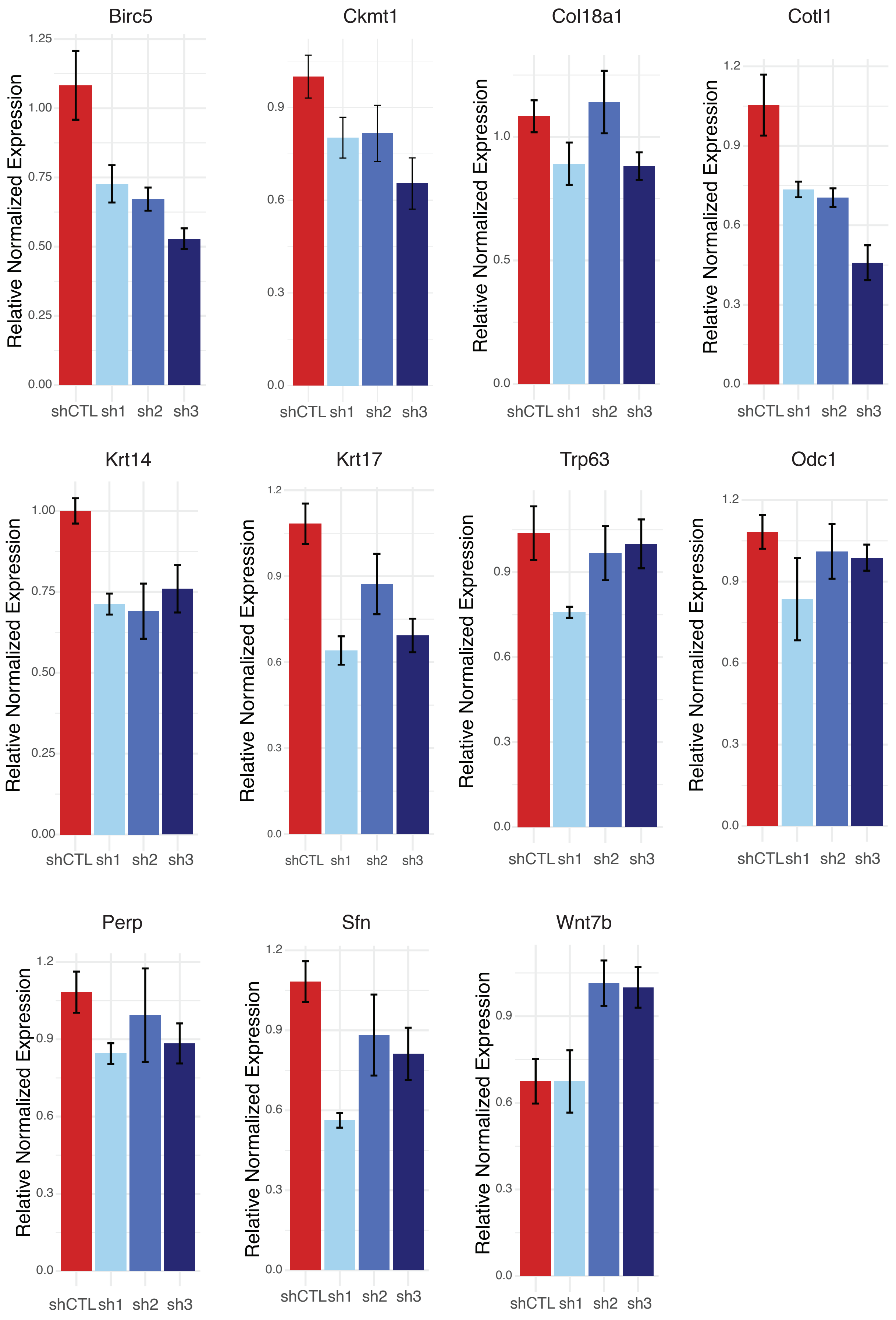

Birc5
Ckmt1
Col18a1
Cotl1
Krt14
Krt17
Trp63
Odc1
Perp
Sfn
Wnt7b
Relative Normalized Expression
shCTL
sh1
sh2
sh3

Figure S3

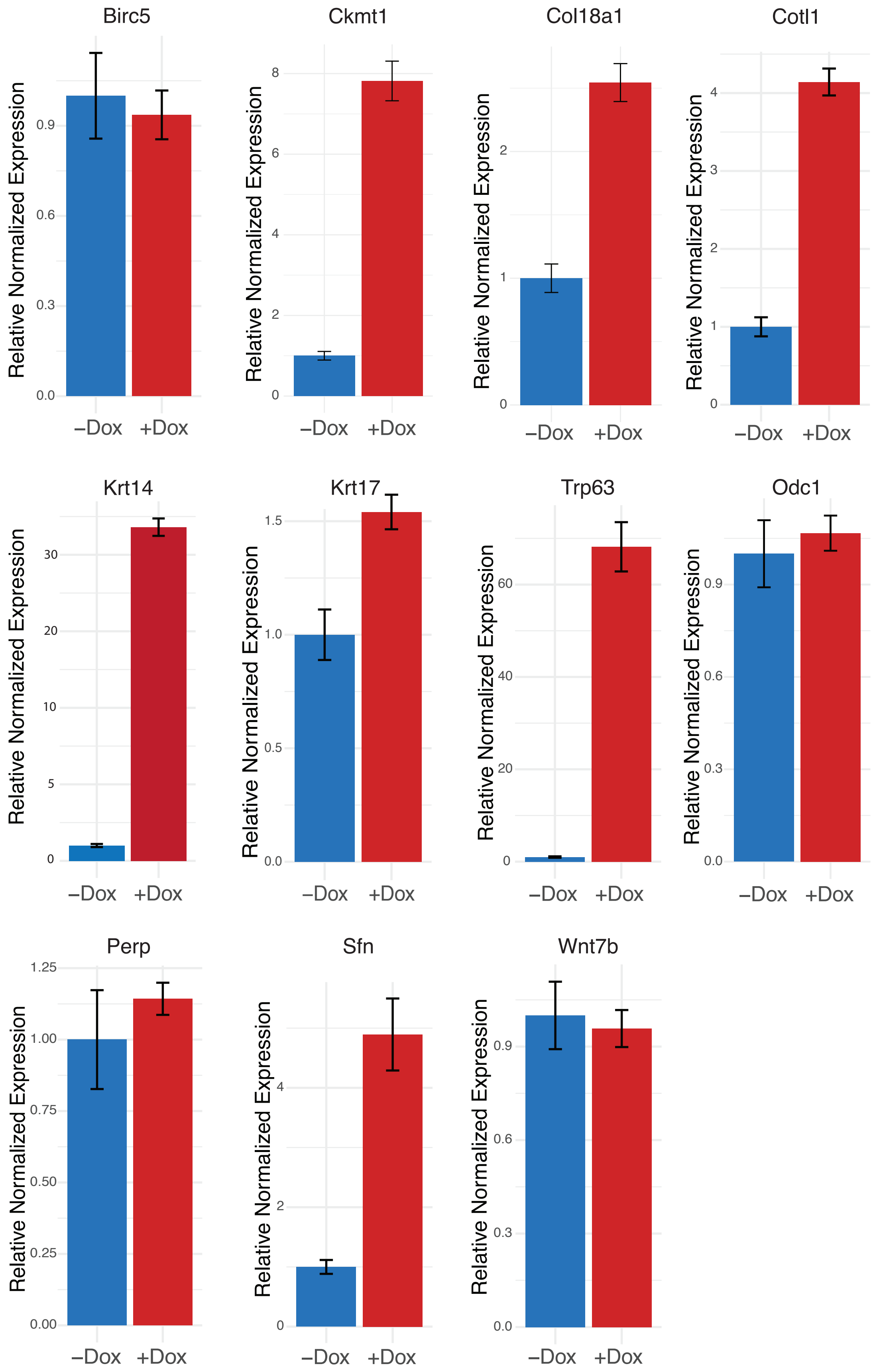

Birc5
Ckmt1
Col18a1
Cotl1
Krt14
Krt17
Trp63
Odc1
Perp
Sfn
Wnt7b
Relative Normalized Expression
−Dox
+Dox

Figure S4

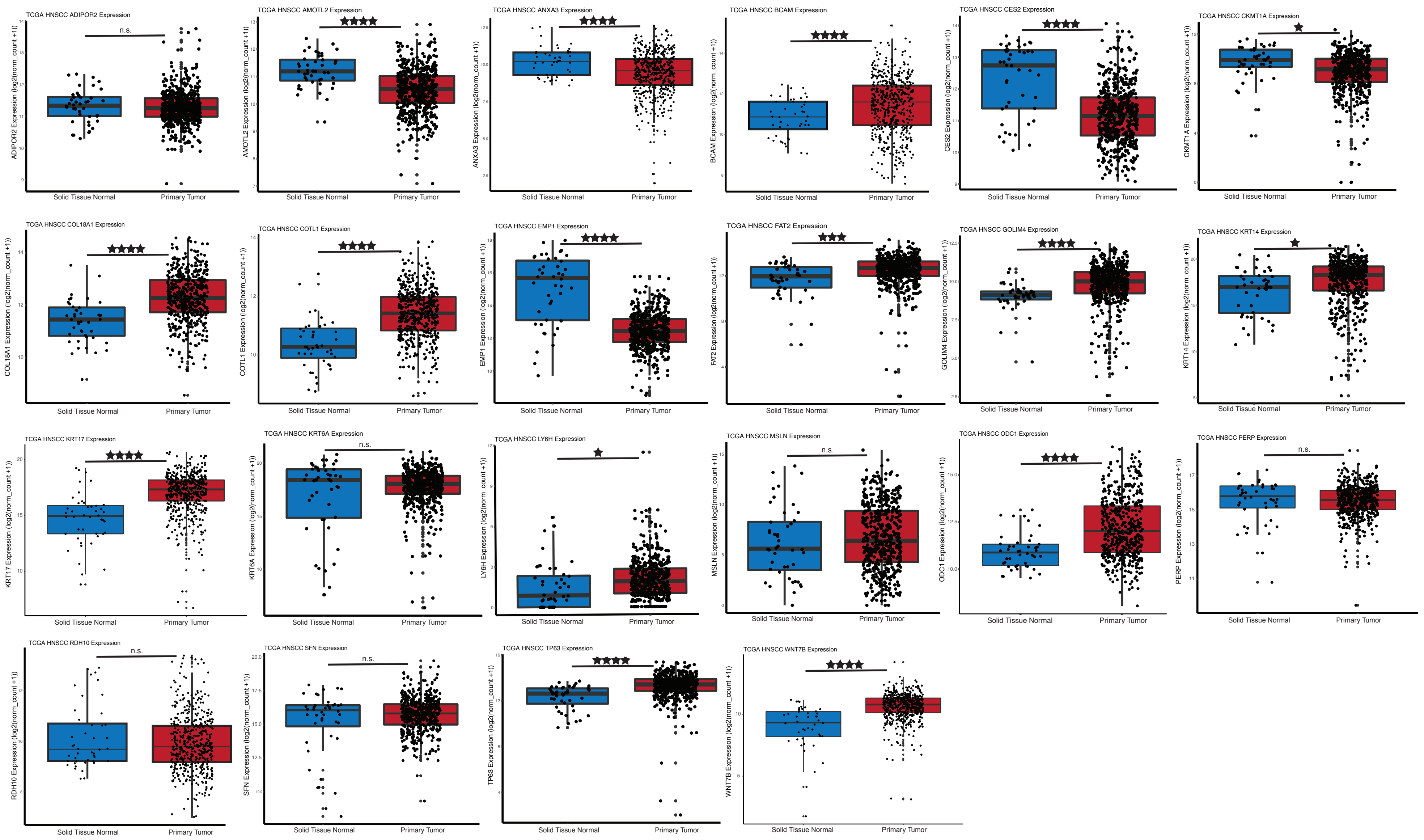
TCGA HNSCC ADIPOR2 Expression
ADIPOR2 Expression (log2(norm_count +1))
n.s.
Solid Tissue Normal
Primary Tumor
TCGA HNSCC AMOTL2 Expression
AMOTL2 Expression (log2(norm_count +1))
Solid Tissue Normal
Primary Tumor
TCGA HNSCC ANXA3 Expression
ANXA3 Expression (log2(norm_count +1))
Solid Tissue Normal
Primary Tumor
TCGA HNSCC BCAM Expression
BCAM Expression (log2(norm_count +1))
Solid Tissue Normal
Primary Tumor
TCGA HNSCC CES2 Expression
CES2 Expression (log2(norm_count +1))
Solid Tissue Normal
Primary Tumor
TCGA HNSCC CKMT1A Expression
CKMT1A Expression (log2(norm_count +1))
Solid Tissue Normal
Primary Tumor
TCGA HNSCC COL18A1 Expression
COL18A1 Expression (log2(norm_count +1))
Solid Tissue Normal
Primary Tumor
TCGA HNSCC COTL1 Expression
COTL1 Expression (log2(norm_count +1))
Solid Tissue Normal
Primary Tumor
TCGA HNSCC EMP1 Expression
EMP1 Expression (log2(norm_count +1))
Solid Tissue Normal
Primary Tumor
TCGA HNSCC FAT2 Expression
FAT2 Expression (log2(norm_count +1))
Solid Tissue Normal
Primary Tumor
TCGA HNSCC GOLIM4 Expression
GOLIM4 Expression (log2(norm_count +1))
Solid Tissue Normal
Primary Tumor
TCGA HNSCC KRT14 Expression
KRT14 Expression (log2(norm_count +1))
Solid Tissue Normal
Primary Tumor
TCGA HNSCC KRT17 Expression
KRT17 Expression (log2(norm_count +1))
Solid Tissue Normal
Primary Tumor
TCGA HNSCC KRT6A Expression
KRT6A Expression (log2(norm_count +1))
n.s.
Solid Tissue Normal
Primary Tumor
TCGA HNSCC LY6H Expression
LY6H Expression (log2(norm_count +1))
Solid Tissue Normal
Primary Tumor
TCGA HNSCC MSLN Expression
MSLN Expression (log2(norm_count +1))
n.s.
Solid Tissue Normal
Primary Tumor
TCGA HNSCC ODC1 Expression
ODC1 Expression (log2(norm_count +1))
Solid Tissue Normal
Primary Tumor
TCGA HNSCC PERP Expression
PERP Expression (log2(norm_count +1))
n.s.
Solid Tissue Normal
Primary Tumor
TCGA HNSCC RDH10 Expression
RDH10 Expression (log2(norm_count +1))
n.s.
Solid Tissue Normal
Primary Tumor
TCGA HNSCC SFN Expression
SFN Expression (log2(norm_count +1))
n.s.
Solid Tissue Normal
Primary Tumor
TCGA HNSCC TP63 Expression
TP63 Expression (log2(norm_count +1))
Solid Tissue Normal
Primary Tumor
TCGA HNSCC WNT7B Expression
WNT7B Expression (log2(norm_count +1))
Solid Tissue Normal
Primary Tumor

Figure S5

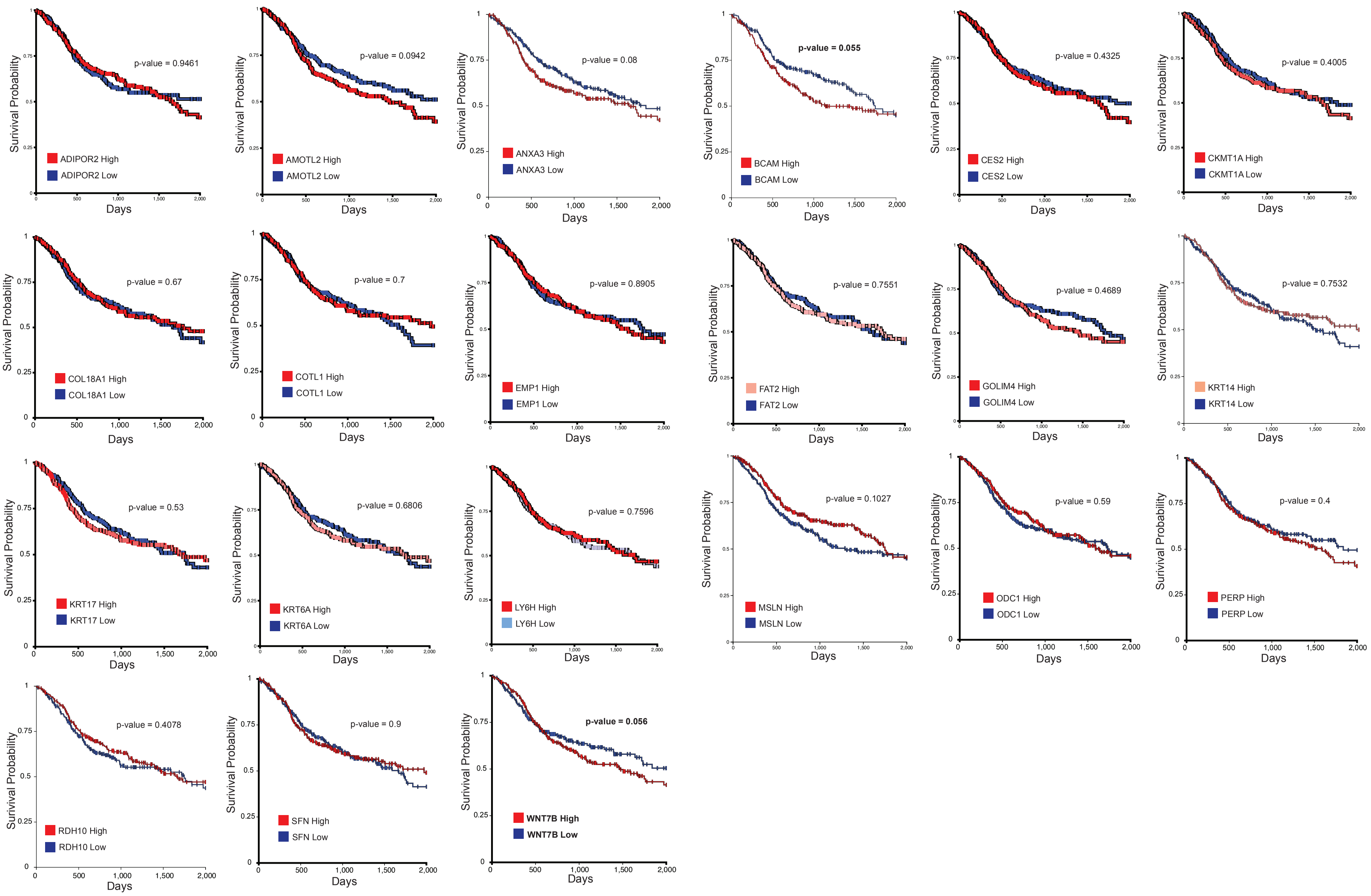

ADIPOR2 High
ADIPOR2 Low
p-value = 0.9461
AMOTL2 High
AMOTL2 Low
p-value = 0.0942
ANXA3 High
ANXA3 Low
p-value = 0.08
BCAM High
BCAM Low
p-value = 0.055
CES2 High
CES2 Low
p-value = 0.4325
CKMT1A High
CKMT1A Low
p-value = 0.4005
COL18A1 High
COL18A1 Low
p-value = 0.67
COTL1 High
COTL1 Low
p-value = 0.7
EMP1 High
EMP1 Low
p-value = 0.8905
FAT2 High
FAT2 Low
p-value = 0.7551
GOLIM4 High
GOLIM4 Low
p-value = 0.4689
KRT14 High
KRT14 Low
p-value = 0.7532
KRT17 High
KRT17 Low
p-value = 0.53
KRT6A High
KRT6A Low
p-value = 0.6806
LY6H High
LY6H Low
p-value = 0.7596
MSLN High
MSLN Low
p-value = 0.1027
ODC1 High
ODC1 Low
p-value = 0.59
PERP High
PERP Low
p-value = 0.4
RDH10 High
RDH10 Low
p-value = 0.4078
SFN High
SFN Low
p-value = 0.9
WNT7B High
WNT7B Low
p-value = 0.056
Surivival Probability
Days
0
0.25
0.5
0.75
1
500
1,000
1,500
2,000

Figure S6

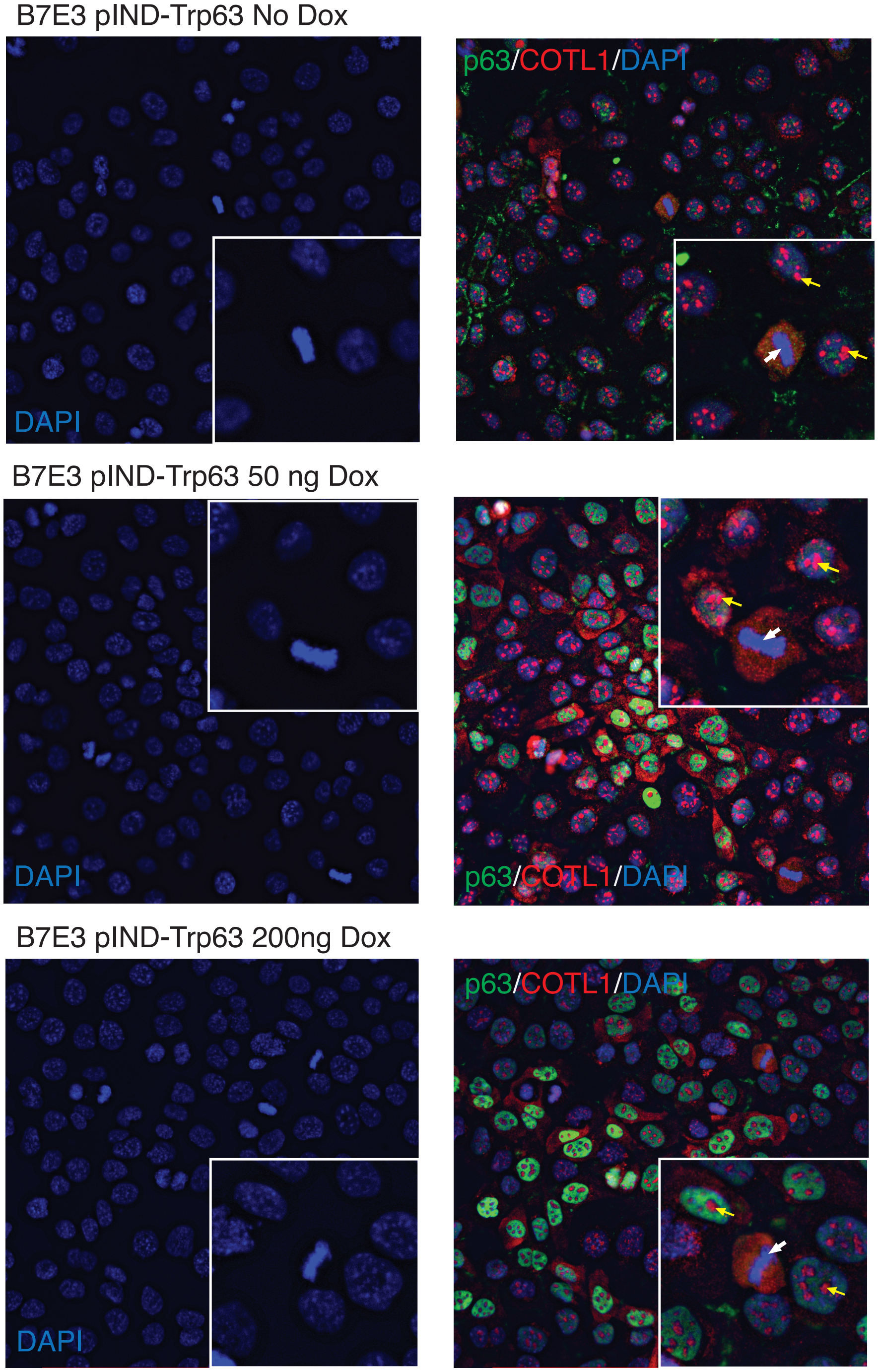
B7E3 pIND-Trp63 No Dox
DAPI
p63/COTL1/DAPI
B7E3 pIND-Trp63 50 ng Dox
DAPI
p63/COTL1/DAPI
B7E3 pIND-Trp63 200ng Dox
DAPI
p63/COTL1/DAPI

Figure S7

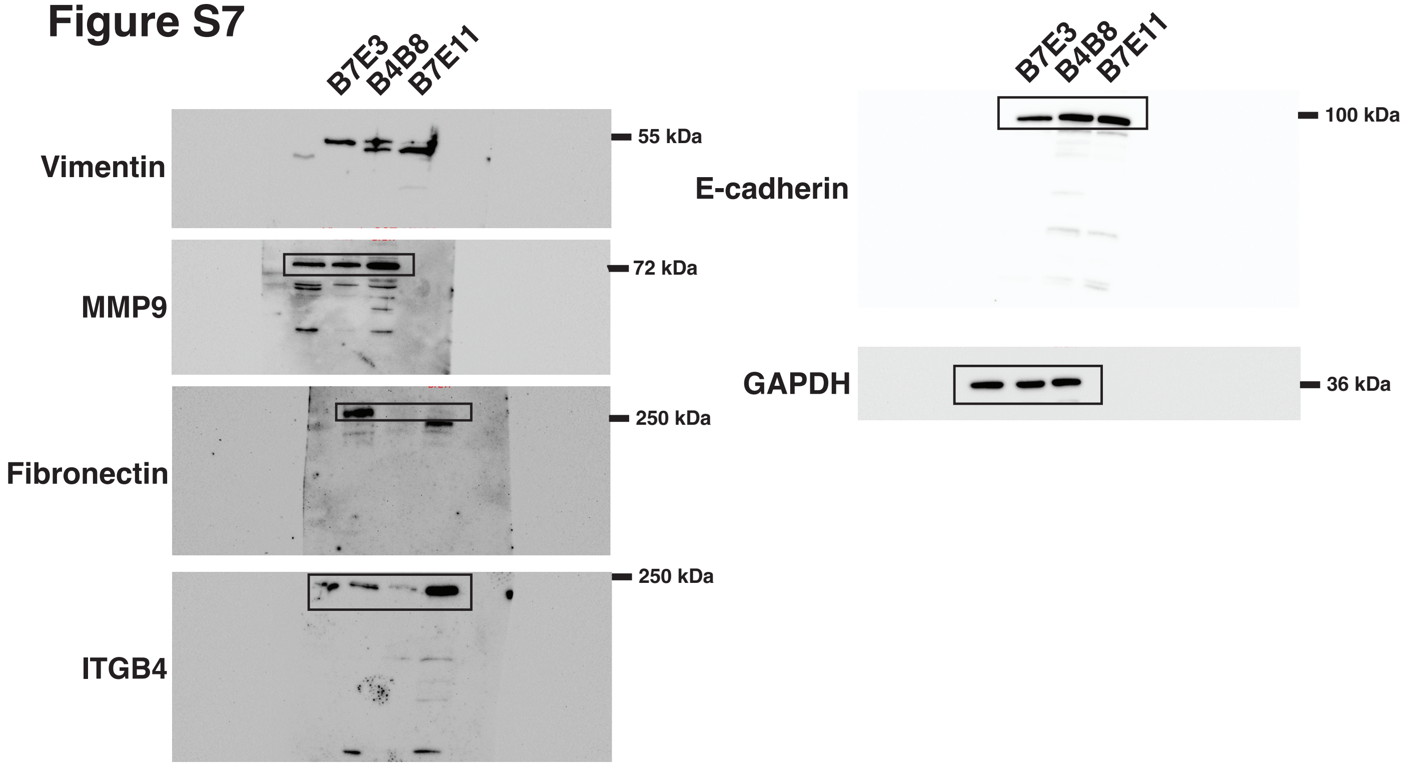

Figure S8

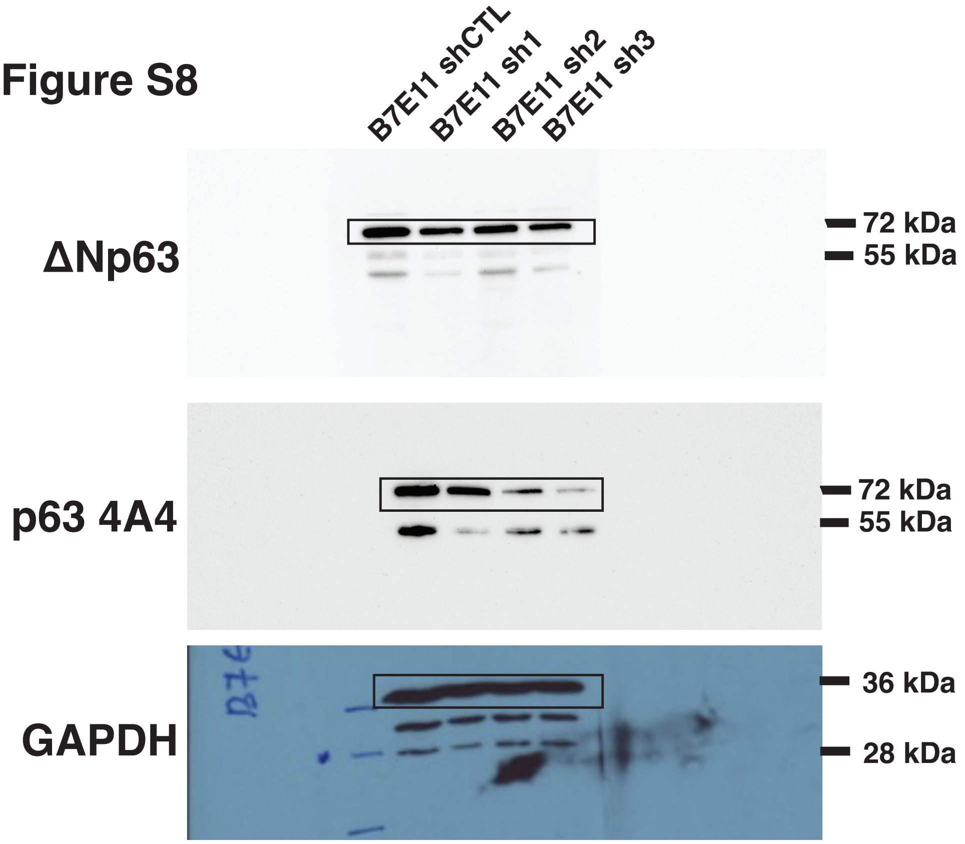

Figure S9

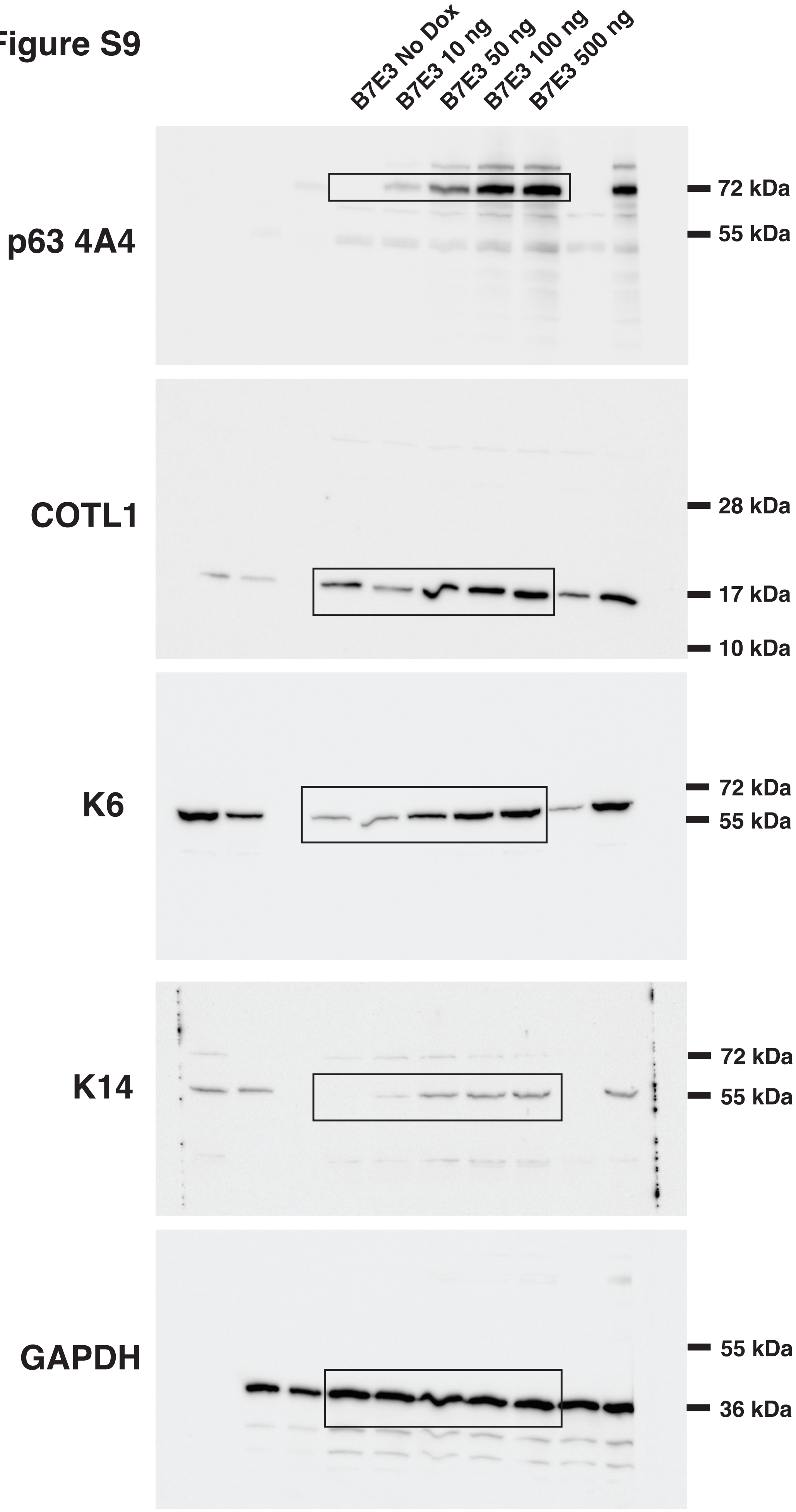

B7E3 No Dox
B7E3 10 ng
B7E3 50 ng
B7E3 100 ng
B7E3 500 ng
p63 4A4
72 kDa
55 kDa
COTL1
28 kDa
17 kDa
10 kDa
K6
72 kDa
55 kDa
K14
72 kDa
55 kDa
GAPDH
55 kDa
36 kDa
